# Learning to Be Elite: Lessons From HIV-1 Controllers and Animal Models on Trained Innate Immunity and Virus Suppression

**DOI:** 10.3389/fimmu.2022.858383

**Published:** 2022-04-27

**Authors:** Sho Sugawara, R. Keith Reeves, Stephanie Jost

**Affiliations:** Division of Innate and Comparative Immunology, Center for Human Systems Immunology, Department of Surgery, Duke University School of Medicine, Durham, NC, United States

**Keywords:** elite controllers (ECs), HIV, innate immunity, NK cells, trained immunity, NHP models

## Abstract

Although antiretroviral therapy (ART) has drastically changed the lives of people living with human immunodeficiency virus-1 (HIV-1), long-term treatment has been associated with a vast array of comorbidities. Therefore, a cure for HIV-1 remains the best option to globally eradicate HIV-1/acquired immunodeficiency syndrome (AIDS). However, development of strategies to achieve complete eradication of HIV-1 has been extremely challenging. Thus, the control of HIV-1 replication by the host immune system, namely functional cure, has long been studied as an alternative approach for HIV-1 cure. HIV-1 elite controllers (ECs) are rare individuals who naturally maintain undetectable HIV-1 replication levels in the absence of ART and whose immune repertoire might be a desirable blueprint for a functional cure. While the role(s) played by distinct human leukocyte antigen (HLA) expression and CD8+ T cell responses expressing cognate ligands in controlling HIV-1 has been widely characterized in ECs, the innate immune phenotype has been decidedly understudied. Comparably, in animal models such as HIV-1-infected humanized mice and simian Immunodeficiency Virus (SIV)-infected non-human primates (NHP), viremic control is known to be associated with specific major histocompatibility complex (MHC) alleles and CD8+ T cell activity, but the innate immune response remains incompletely characterized. Notably, recent work demonstrating the existence of trained innate immunity may provide new complementary approaches to achieve an HIV-1 cure. Herein, we review the known characteristics of innate immune responses in ECs and available animal models, identify gaps of knowledge regarding responses by adaptive or trained innate immune cells, and speculate on potential strategies to induce EC-like responses in HIV-1 non-controllers.

## Introduction

Despite the success of antiretroviral therapy (ART), completely eradicating human immunodeficiency virus-1 (HIV-1) from people living with HIV-1 (PLWH) remains extremely challenging due to long-lived HIV-1 latent reservoirs (1, 2). Instead, strategies based on the control of HIV-1 replication by host immune responses have long been investigated to establish a functional cure for HIV-1. HIV-1 elite controllers (ECs) are infrequent cohorts who naturally maintain undetectable HIV-1 replication levels in the absence of ART and whose immune responses provide a model for functional cures (3). Numerous studies uncovered effective CD8+ T cell responses associated with specific human leukocyte antigen (HLA) representation in ECs (4–6), yet attempts to induce similar protective T cell responses in other PLWH have not been adequately successful (7, 8). Conversely, innate immune responses in ECs have mostly been understudied. Because innate immune cells orchestrate adaptive immune responses in multiple diseases (9, 10), understanding innate responses in ECs could open a new avenue to improve CD8+ T cell immunity in HIV-1 non-controllers and achieve functional cure. Moreover, along with canonical innate responses, enhanced innate immunity upon repeated pathogen exposures, collectively referred to as trained immunity, was recently described against several pathogens (11–13). Trained immunity was also reported to modulate simian immunodeficiency virus (SIV)/HIV-1 control (11, 14–16), further supporting the potential important contribution of innate immune responses during HIV-1 infection in ECs. Because ECs represent an extremely limited population of PLWH, utilizing animal models including humanized mice and SIV-infected non-human primates (NHP) would allow rigorously investigating distinct conventional and trained innate immune responses associated with elite control of HIV-1 (17). In this review, we exhaustively elucidate the known characteristics of innate immune responses in ECs, highlight available animal models and their innate immunity, discuss the gaps in knowledge on recall responses in adaptive and innate immunity, and explore the potential strategies to elicit EC-like responses in both PLWH and animal models.

## HIV-1 Elite Controllers and Their Adaptive Immune Responses

HIV-1 controllers are traditionally classified based on CD4 counts and viral load. Long-term non-progressors (LNTPs) represent a subpopulation of PLWH who can sustain CD4 counts of more than 500 cells/μl of blood for longer than 7 years after infection (3, 18, 19). Unfortunately, LNTPs occupy only 2% of PLWH. ECs represent a further restricted cohort amongst LNTPs (0.3%) who can maintain undetectable viral loads (less than 50 copies/ml) in addition to stable CD4 counts for more than 12 months without ART (3, 18, 19). Individuals with plasma HIV-1 RNA levels of 50 to 2,000 copies/ml are often defined as viremic controllers (VCs) in comparison to ECs (20). The viremic control of ECs is considered as temporary because only 1% of them can maintain their virological control for more than 10 years (3). While ECs harbor HIV-1 reservoirs that are more transcriptionally silent than those in HIV-1 non-controllers (21), it is well appreciated that viruses isolated from ECs can replicate as robustly as those from viremic individuals (22). Rather, host immune responses are likely the major contributor of spontaneous control (3, 23–25). One of the hallmarks of EC immune responses is their strong CD8+ T cell responses that are associated with viremic control (23, 26, 27). Correspondingly, many groups reported that specific alleles of HLA class I molecules, particularly HLA-B*27 or B*57, are over-represented in ECs (4–6). The stronger CD8+ T cell responses can also be attributed to the difference in their CD4+ T cell subsets (3, 28). While reports are varied on the susceptibility of EC CD4+ T cells to HIV-1 infection (29, 30), ECs consistently maintain the balance of CD4+ T helper (Th) 17 cell and regulatory (Treg) subsets similar to that of HIV-1 uninfected individuals, while the ratio of Th17/Treg cells is lower in viremic subjects (31, 32). Whereas robust T cell activity is often observed in ECs, their B cell responses do not seem to significantly contribute to their viremic control. Rather, ECs exerted weaker neutralizing and non-neutralizing antibody responses than VCs (33, 34). Nonetheless, because protective HLA genotypes do not always confer EC phenotypes (6), it is highly plausible that other subsets of immune cells, particularly innate effector cells, could also contribute to the spontaneous control in ECs.

## Innate Immune Responses in HIV-1 Elite Controllers

### NK Cells

Whereas studies on HIV-1 ECs have intensively focused on adaptive immunity due to the association with HLA-B molecules, it is possible that innate immune responses are also involved in viremic control (**Table 1**). Indeed, ECs exhibit increased markers of inflammation such as elevated interferon (IFN)-stimulated gene expression (47), suggesting the activation of innate immune responses. Among innate immune cells, natural killer cells (NK cells) are critical effector cells that play an important role in HIV-1 infection (50–52). Killer immunoglobulin-like receptors (KIRs) on NK cells are key receptors that regulate NK cell functions and several KIR have been associated with control of HIV-1 (53). HLA-B*57-01 is known to interact with NK cells *via* KIR3DL1 and KID3DS1+ NK cells can suppress viral replication (35, 54–56). As expected, HIV-1 viremic control is positively correlated with percentage of NK cells (38) and HIV-1 protection is associated with specific KIR3DL1 allotypes in HLA-B*57-positive subjects (57). Additionally, NK cells from ECs can efficiently lyse HIV-1-infected CD4+ T cells independently of KIR3DS1 expression (36), suggesting other factors yet to be defined influence their unique antiviral activity. Moreover, the percentage of dysfunctional CD56-CD16+ NK cells, which are frequently observed in PLWH, was lower in ECs than viremic subjects (25). EC NK cells also express higher levels of the activating receptor NKp46 (37) and secrete more IFNγ (38), but their antibody-dependent cellular cytotoxicity (ADCC) responses were not found elevated compared to non-controllers (33). Altogether, these observations indicate that NK cell responses may be enhanced in ECs.

**Table 1:** Characteristics of HIV-1 elite controller innate immune cells.

Innate immune cells	Characteristics in ECs
**NK cells**	•Association of KIR haplotypes with HIV-1 protection (35) •Robust cytotoxicity against target cells (36) •Lower percentage of defective CD56-CD16+ cells (25) •Increased activating receptor expression (37) •Upregulated IFNγ secretion (38)
**ILCs**	•Maintenance of all ILC subsets in PBMCs (39)
**cDCs**	•More circulating cDCs (40, 41) •Elevated cGAS signaling by HIV-1 stimulation (42) •More autologous T cell activation induced (42) •Increased receptor expression for antigen capture (43)
**pDCs**	• More in circulation than in viremic individuals (38, 44, 45) • Secrete equivalent levels of type 1 IFN to uninfected people (38, 44, 45) • Intracellular-specific TRAIL expression (45) • Equivalent gut homing marker expression to uninfected individuals (24)
**Monocytes**	• Lower percentages of CD14++CD16+ monocytes (46) • Weaker responses to LPS stimulation (47)
**Macrophages**	• Similar HIV-1 susceptibility to viremic individuals (48)
**Granulocytes**	•Upregulated antiviral factor expression by HIV-1 stimulation (40) •Better survival of neutrophils cultured in supernatant from HIV-1 infected PBMCs (49)

The distinguished hallmarks of each innate immune cell in HIV-1 elite controllers are tabulated. ECs, elite controllers; NK, natural killer; ILCs, innate lymphoid cells; DCs, dendritic cells; cDCs, conventional dendritic cells; pDCs, plasmacytoid dendritic cells; IFN, interferon.

### Innate Lymphoid Cells

Similar to NK cells, innate lymphoid cells (ILCs) are increasingly studied innate immune subsets that could potentially be altered in ECs. ILCs are lymphoid-lineage cells that are distinct from T cells and B cells and display early responses to pathogens or tissue injuries (58, 59). Depending on the expression of transcription factors, ILCs are further classified as ILC1, ILC2, and ILC3 and their functions appear to mirror those of CD4+ T helper (Th)1, Th2, and Th17 lymphocytes, respectively (58). ILC1 include NK cells, which can be viewed as the innate counterpart of CD8+ T cells. Each subset of ILCs secretes distinct cytokines. ILC1 secretes IFNγ, and ILC2 produces IL-5 and IL-13. ILC3 is the major producer of IL-17 and IL-22 (58). In PLWH, ILC depletion was not observed in mucosal tissues (39), yet all subsets of circulating ILCs were depleted during chronic HIV-1 infection, presumably by over-activation of ILCs (39, 59, 60). Cytokine production by ILC1 was also impaired in PLWH (59). Intriguingly, Kloverpris et al. (39) reported that treatment naïve aviremic PLWH did not experience ILC depletion though they did not specify whether their non-viremic PLWH are ECs or not. Thus, the role played by ILCs in elite control of HIV-1 remain unclear. However, considering data from PLWH, it is plausible that ECs maintain circulating ILC populations and their ILCs secrete cytokines more robustly than HIV-1 non-controllers, which may be one contributing factor for elite control.

### Dendritic Cells

Dendritic cells (DCs), which are one of the critical immune cells interacting with NK cells and T cells, also have differential signatures in ECs. Two main subsets of DCs can be found in the peripheral blood: conventional DCs (cDCs) and plasmacytoid dendritic cells (pDCs) (61). cDCs primarily present antigens to T cells and thereby modulate adaptive immunity (61). In HIV-1 infection, more cDCs are observed in circulation in ECs than in viremic subjects (40, 41). Besides being more abundant, cDCs also exert improved immune responses in ECs. cDCs in ECs induce more cGAS signaling molecule expression upon HIV-1 stimulation, and as a consequence secrete more type 1 IFN (42). EC cDCs also undergo quicker maturation after stimulation by HIV-1 (42). Furthermore, EC cDCs express more surface receptors critical for capturing HIV-1 antigen (43) and modulating other immune cells (62). Consequently, EC cDCs more effectively activate autologous CD4+ and CD8+ T cells (42).

Another subset of DCs, pDCs, also differ in ECs. pDCs are the major producer of type 1 IFN (63), and their numbers decrease in all PLWH (44). However, in ECs pDC numbers were higher than in viremic subjects and secreted abundant type 1 IFN similar to healthy donors (38, 44, 45). Interestingly, the expression of gut-homing marker α4β7 is elevated in both ECs and HIV-1 non-controllers compared to HIV-1 uninfected individuals (24). This indicates the loss of circulating pDCs in PLWH plausibly results from increased gut trafficking rather than depletion of peripheral blood pDCs. Additionally, Barblu et al. (45) demonstrated healthy donor and EC pDCs only had intracellular TRAIL expression, a ligand for apoptosis-inducing receptors (64), while both surface and intracellular TRAIL expression were exhibited in pDCs from HIV-1 non-controllers. They also co-cultured pDCs from EC and viremic individuals with HIV-1 chronically-infected CD4+ T cell line H9 and reported that co-culture with EC pDCs induced more apoptosis of H9 cells (45). However, it is difficult to speculate whether pDCs similarly trigger apoptosis of HIV-1-infected primary CD4+ T cell from this observation alone.

### Monocytes and Macrophages

Monocytes are another indispensable innate effector cell that exhibits unique characteristics in ECs. HIV-1-associated neurocognitive disorders (HAND) likely result from chronic inflammation of the central nervous system and correlate with monocyte activation. Accordingly, monocytes from PLWH experiencing HAND produced more inflammatory cytokines than those from PLWH without HAND (65). These enhanced monocyte responses can be due to perturbations of the monocyte compartment. Chen et al. (66) reported that chronic HIV-1 infection increased the percentage of intermediate monocytes (CD14++CD16+ cells) in blood, which exert inflammatory responses (67). Within this subset, CD163+CD16+ monocytes exhibited a negative correlation with CD4+ T cell counts (68). Conversely, ECs are known to experience weaker neuroinflammation compared to HIV-1 non-controllers (69), which reflects more preserved proportions of the different monocyte subsets. ECs accumulated lower percentages of intermediate monocytes compared to viremic subjects (46) and their monocytes triggered weaker responses to LPS stimulation than those from ART-suppressed PLWH (47).

In addition to monocytes, macrophages are critical phagocytic cells as well as target cells for HIV-1 infection. Many groups have reported that HIV-1 persists in monocyte-derived macrophages (MDMs) and tissue-resident macrophages (70–72). Regarding their susceptibility to HIV-1 infection, Walker-Sperling et al. (48) demonstrated no significant differences in MDMs from ECs and viremic subjects. Macrophages also exert phagocytosis and secrete proinflammatory cytokines in response to HIV-1 infection (73). However, phagocytic activity is altered in both HIV-1-infected and uninfected bystander macrophages (74, 75). In ECs, it has not been investigated yet as to whether macrophages more robustly produce cytokines and restore their phagocytic activities, which are impaired in HIV-1 viremic subjects.

### Granulocytes

Along with macrophages, granulocytes, such as neutrophils, basophils, and eosinophils, are other innate immune cells modulated by HIV-1 infection. Jiang et al. (76) reported that basophils can capture HIV-1 virions and facilitate HIV-1 transmission to CD4+ T cells, and increased eosinophil counts are frequently observed in PLWH (77), highlighting the potential roles of granulocytes in HIV-1 infection. As anticipated, EC granulocytes exhibited elevated expression of antiviral factors when stimulated with HIV-1 (40). Neutrophils from ECs also demonstrated a better survival than those from non-controllers when cultured with GM-CSF secreted from HIV-1-infected PBMCs (49), but their antibody-dependent phagocytosis was not different from that in HIV-1 non-controllers (33). Though their intrinsic immune functions may not be upregulated in ECs, several studies indicate the potential alteration of other immune cells by neutrophils. Neutrophils in PLWH express more Programmed Death-Ligand 1 (PD-L1), thereby suppressing CD8+ T cell responses (78). This implies that neutrophils in ECs could regulate other immune cells in a unique fashion. Unfortunately, little research has been performed to investigate the distinct characteristics of basophils and eosinophils in ECs. However, given the roles of these immune cells in HIV-1 transmission and pathogenesis, it is plausible that EC basophils and eosinophils also exhibit distinguishable surface receptor expression and functional responses compared to non-controllers.

In summary, innate immune cells in ECs exhibit unique features compared to other PLWH, although the studies on certain innate effector cells are still limited. Available data imply that ECs can successfully maintain the balance of pro-inflammatory and anti-inflammatory responses elicited by respective innate immune subsets. Although it has not been elucidated yet, the maintenance of this innate immune balance seems to be indispensable for exerting robust HIV-1 specific responses by both innate and adaptive immune cells. Given their roles in modulating T cell responses (9, 10, 78), it would be important to study EC innate immunity in greater detail to further elucidate their contributions to improved CD8+ T cell responses. However, because of the rarity of this population, research solely depending on available EC samples may not be practical to exhaustively delineate innate immune responses associated with natural control of HIV-1.

## Animal Models for HIV-1 Elite Controllers

### Humanized Mice

Because HIV-1 elite controllers represent a minor portion of PLWH, it would be beneficial to establish animal models such as humanized mice and SIV-infected NHP with immune responses that mirror those of elite controllers. In the humanized mouse model, the immune system is reconstituted with human immune cells (79). Whereas mice are not susceptible to HIV-1 infection, numerous groups confirmed that HIV-1 can replicate in various humanized mice models (80–83), which are widely used to examine promising immunotherapies (84), latency reversing agents (85), and gene-editing of HIV-1 proviruses *in vivo* (86, 87). This model has been criticized for frequent development of graft versus host disease (GvHD) symptoms affecting studies of innate immunity, yet recent models such as C57BL/6 RAG2 -/- common γ chain -/- CD47 -/- triple knockout mice present dramatically reduced risks of GvHD and improved reconstitution of human adaptive immune system (88).

In HIV-1 infected humanized mice, ILC1 and ILC3 are depleted in lymphoid tissues by type 1 IFN produced by pDCs (59, 60). Recently, Kim and colleagues (89) demonstrated that infusion of allogenic human NK cells in HIV-1-infected humanized mice can delay HIV-1 viral rebound after ART interruption. Additionally, animals who received allogenic NK cell transfer displayed reduced diversity in HIV-1 species, highlighting the significance of NK cells in HIV-1 control in this model. In order to investigate EC immune responses *in vivo*, Dudek et al. (90) generated humanized BLT (bone marrow, liver, thymus) mice, where implanted human fetal CD34+ hematopoietic stem cells (HSCs) become educated within transplanted autologous human thymic tissues, using HSCs expressing either the protective HLA-B*57 or non-protective HLA-B alleles. Although mice reconstituted with HLA-B*57 positive cells demonstrated better control of HIV-1 replication, they could not suppress HIV-1 replication to the point where viral loads would be lower than the limit of detection, in contrast to human ECs. It is important to note that their humanized mice model poorly reconstitute innate immune cells (79), and robust CD8+ T cell responses have been positively associated with more successful reconstitution of monocytes in humanized mouse models (91). Therefore, one possible explanation for why humanized mice could not achieve HIV-1 control is that EC innate immune responses were not recapitulated in their model. Recent humanized mouse models such as MISTRG mice drastically improved the reconstitution of diverse subsets of innate immune cells by replacing murine cytokine genes for human homologs (92, 93). It would be intriguing to compare the immune responses between traditional and next generation humanized mice models reconstituted with EC immune systems to investigate how innate immunity contributes to natural control of HIV-1.

### Non-Human Primates

SIV-infected non-human primates, including rhesus macaques (*Macaca mulatta*), African green monkeys (AGM) (*Chlorocebus aethiops*), pig-tailed macaques (*Macaca nemestrina*) and Mauritan cynomolgus macaques (MCM) (*Macaca fascicularis*), are well-established animal models for HIV-1 research that have been valuable for studying innate immunity. Elevated levels of inflammatory cytokines such as IL-6 and type 1 IFN are observed in numerous SIV infection models (94, 95). Specifically, chronic upregulation of type 1 IFN levels in the blood is linked to pathogenic SIV infection (95, 96). As expected, this pro-inflammatory cytokine environment is associated with modulation of innate immune cells during SIV infection. First, contrary to what was reported in human studies, subsets of ILCs such as NKp44+ and IL-17+ ILCs are depleted in the intestinal mucosa (97, 98). Second, NK cell activities are also downregulated in pathogenic SIV infection (99). NKp44+ NK cells in the gut were significantly depleted during persistent SIV infection, and the magnitude of NKp44+ NK cell depletion was strongly correlated with intestinal CD4+ T cell loss (100). NK cells also lost expression of multiple lymph node trafficking receptors in pathogenic SIV infection and consequently, their homing to the lymph nodes was diminished (101). Finally, increased infiltration of inflammatory monocytes into the liver was demonstrated in SIV-infected rhesus macaques and was associated with hepatic viral replication and markers of liver inflammation (102). Altogether, SIV infection exhibits numerous hallmarks of inflammation that lead to the dysfunction of multiple innate immune subsets.

A number of SIV-infected EC NHP models were considered to study human EC-like innate responses. One strategy to establish SIV elite controllers is to infect macaques with an SIV strain from other NHP species, such as an AGM strain, or with a mutated SIV. Pandrea et al. (103) reported that rhesus macaques infected with the AGM strain of SIV controlled viral replication at the later stage of infection, and CD8+ T cells were critical for suppressing viremia. Breed and colleagues generated an SIV strain without the GYxxO cytoplasmic trafficking motif in Env (SIV ΔGY) that transiently infects gut CD4+ T cells and therefore does not deplete intestinal CD4+ T cells (104). Using this strain, they demonstrated that infection of pig-tailed macaques also resulted in spontaneous control similar to HIV-1 ECs. These animals experienced reduced monocyte depletion, which is one of the hallmarks of pathogenic infection (105). However, it is important to note that viral replication and transmission kinetics of these strains in macaque species are likely to differ from those of wild type SIV (103, 105). As numerous studies have reported the equivalent replication capacity between EC and non-controller viruses (22), infection with mutated SIV or viruses adapted to other primate species may not be an appropriate representation of immune control of HIV-1 in ECs.

Similar to HIV-1 elite controllers, viremic control in SIV infection is associated with specific Major Histocompatibility Complex (MHC) alleles in rhesus macaques. Therefore, another approach to study elite control of SIV is to infect macaques expressing protective MHC with SIV. Loffredo et al. (106) demonstrated that rhesus macaque MHC molecules Mamu-B*03 and Mamu-B*08 bind epitopes similar to human HLA-B*27, and animals bearing these MHC alleles were more likely to establish spontaneous control of SIV infection (107). Other studies with rhesus macaques also indicated the association of Mamu-B*17, Mamu-B*1001 and Mamu-B*8701 with viremic control (108, 109). As anticipated, robust CD8+ T cell responses were associated with viremic control in these models similar to human ECs (107, 110). Intriguingly, SIV EC rhesus macaques had higher percentage of plasmacytoid dendritic cells in circulation similar to human ECs, but lower in colorectal tissues than animals with high viremia (38, 111). Correspondingly, more IFNα-positive pDCs were observed in PBMCs from SIV elite controllers than viremic animals, but this trend was reversed in colorectal samples (111). Because SIV infection is known to promote robust inflammatory responses (112), it would be intriguing to assess the magnitude of systemic inflammation in SIV EC animals.

Besides the rhesus macaque model, MCM models have been frequently used to recapitulate immune responses in human ECs. MCMs with SIV infection exhibit similar phenotypes as human ECs including lower viral loads and reduced CD4+ T cell loss (113, 114). Similar to other animal models, MCMs expressing specific MHC haplotypes such as M1, M2, and M6 are likely to establish viremic control (114–116). Surprisingly, several studies indicate that effective CD8+ T cell responses are not required for viremic control in this model (113, 114), suggesting the potential contribution of innate immune cells in HIV-1/SIV elite control.

Although MCM models indicate that elite control can potentially be attributed to innate immunity, the majority of innate immune cells has been understudied in both rhesus and cynomolgus macaque models in the context of elite control. SIV-infected NHP models have many advantages over human studies including the ability to experimentally deplete specific immune cells (117), the availability of tissue samples (117, 118), and the carefully-controlled design of the studies with clear definition of acute and chronic infection (118). It would be beneficial to investigate whether specific depletion of innate effector cells, such as monocytes, neutrophils, and NK cells, would alter the outcome of SIV infection in macaques expressing protective MHC alleles. Furthermore, SIV EC models could unravel the distinguishable innate immune responses in different anatomical sites, which is challenging to demonstrate in human ECs.

## How to Induce HIV-1 Elite Controller Responses?

Therapeutic vaccines that elicit EC-like responses have been investigated in order to achieve a functional cure in PLWH with diversified MHC genotypes. In SIV-infected monkeys expressing Mamu-B*08, vaccination with Mamu-B*08-restricted peptides induced robust CD8+ T cell responses (119). Migueles et al. (7) tested adenovirus-based vaccine in HIV-1 controllers and non-controllers and investigated their CD8+ T cell responses. Unfortunately, only HLA-B*57-positive HIV-1 non-controllers could induce highly functional CD8+ T cell activities similar to ECs. Additionally, Li et al. reported a more effective reduction in HIV-1 reservoir size following vaccination in ECs compared to non-controllers though it was not statistically significant (120). This study also showed an inverse correlation between HIV-1 reservoir size and percentage of activated CD8+ T cells, suggesting that the decrease in EC HIV-1 reservoir size may be partially mediated by CD8+ T cell activities. Whereas T cell-based therapies can induce robust HIV-1 specific responses in ECs, these strategies need to be further ameliorated by activation of other subset of immune cells including innate effector cells so that effective HIV-1-specific responses can be globally triggered in PLWH regardless of their diverse MHC haplotypes.

Chimeric antigen receptor (CAR) therapies are emerging immunotherapeutic approaches that could engineer innate cellular immune products that mimic responses in ECs. A CAR is comprised of a single chain portion of the variable domain of antibodies (scFv) and of an intracellular signaling domain such as CD3 ζ chain and Fc receptor γ chain (FcRγ) (121). While CARs were initially developed for T cell immunotherapeutics (121), the same concept has more recently been applied to innate effector cells including NK cells (122), macrophages (123), and dendritic cells (124). Specifically, NK cells have been the most intensively investigated among innate immune cells for the application of CAR therapy because of their success in tumor treatment where they exhibit robust responses against lymphoid tumors with limited adverse effects (122). Subrakova et al. (125) introduced the knockout of inhibitory signaling molecule SHP2 in the CAR YT cell line which ameliorated the cytolytic function (125). This implies that CAR-NK therapy can be further complemented by gene knockout and overexpression. Unfortunately, CAR-NK cell therapy has not been tested in PLWH yet (126), but adoptive transfer of CAR-NK cells eliciting EC-like responses could be a promising strategy to establish HIV-1 elite control.

Harnessing trained innate immunity represents another way that could complement the existing approaches to trigger EC-like responses. Instead of genetic recombination, trained immunity by innate effector cells is developed through epigenetic or transcriptional reprogramming (11, 127). Trained immunity can develop upon direct exposure to pathogens, or indirectly *via* pathogen-associated molecular patterns and cytokine milieu generated by host immune responses against pathogens (127). This concept is supported by multiple lines of recent evidence. For instance, *Mycobacterium bovis* Bacillus Calmette-Guérin (BCG)-trained monocytes responded more robustly to LPS stimulation than naïve monocytes (12). Enhanced immunity by trained monocytes depends on H3K4 trimethylation. Macrophage responses were also ameliorated by repeated antigen stimulation from *Nippostrongylus brasiliensis*, and neutrophils are crucial to facilitate enhanced macrophage activities (13). Jensen and colleagues (14) investigated the effect of trained immunity against SIV infection by challenging animals with BCG prior to SIV infection. Animals exposed to Mtb and BCG displayed augmented activation of monocytes after SIV infection, but also enhanced CD4+ T cell activation that could potentially result in higher susceptibility to SIV infection. In HIV-1 ECs, it remains to be determined whether myeloid cells exhibit reduced trained immunity. Nevertheless, as demonstrated by Jensen et al. (14), non-specific induction of trained immunity may not be an effective approach for HIV-1 elite control due to possible off-target effects on other immune cells. Instead, infusion of specific innate effector cells exhibiting enhanced immunity such as CAR expression, knockout of anti-inflammatory genes, and overexpression of innate effector molecules could be a more feasible strategy to utilize trained immunity for HIV-1 immunotherapeutics.

Among innate effector cells, trained immunity exerted by specific subsets of NK cells, namely adaptive NK cells, is increasingly described against several pathogens in humans and animal models. Distinct types of adaptive or memory NK cells have been identified and include cytokine-induced, FcRγ-deficient (Δg), and antigen-specific NK cells (128). Cytokine-induced memory-like (CIML) NK cells mediate enhanced effector functions upon cytokine or activating receptor re-stimulation for several weeks following short-term pre-activation with IL-12, IL-15 and IL-18 (128–130). Owing to their robust anti-tumor responses, CIML NK cells are increasingly studied as a promising target in cancer immunotherapy (131). CIML NK cells exerted effective cytotoxicity against acute myeloid leukemia (AML) and ovarian cancer cells both *in vitro* and *in vivo* mice model (132, 133). Romee et al. (132) performed an allogenic transfer of CIML NK cells in individuals with AML. CIML NK cells exhibited robust expansion and effective anti-tumor responses in the recipients, resulting in a 55 percent overall response rate (132). In viral infection, CIML NK cell responses were observed in individuals post influenza vaccination, with IL-2 being critical for enhanced NK cell responses (134). Despite the promising results on CIML NK cell activity in a number of disease models, little is known about their significance in HIV-1 infection and responses in individuals vaccinated with HIV-1 antigens. It would be intriguing to elucidate the importance of CIML NK cell responses in HIV-1 controllers and investigate the potential use of CIML NK cells as immunotherapeutics for HIV-1 cure.

Gamma signaling chain-deficient (Δg) NK cells are another subset of adaptive NK cells that are specialized in antibody-mediated responses. Δg NK cells often exhibit reduced FcRγ and Syk expression (135) and demonstrate differential surface receptor expression profile including diminished TIM-3 and CD7 expression and increased CD2 expression (135, 136). The loss of Syk expression in Δg NK cells is mediated by epigenetic modification of the Syk promoter region, exhibiting a hallmark of trained immunity (135). This subset of cells expands in individuals with human cytomegalovirus (HCMV) infection, and rhesus macaques with rhesus CMV infection (135, 137). Functionally, Δg NK cells elicited elevated ADCC responses compared to conventional NK cells (135), whereas killing of target cells triggered by other activating receptors was diminished (138). In HIV-1 infection, this subset of NK cells expanded in viremic and ART-suppressed PLWH, and Δg NK cells exerted stronger responses mediated by antibodies (16). Unfortunately, the contribution of Δg NK cell responses in ECs has not been exhaustively elucidated yet. A recent study by Liu et al. (139) illustrated that knockout of FcRγ in human NK cells enhanced cytokine secretion by CD16 stimulation while cytotoxic responses mediated by other activating receptors were downregulated, similar to Δg NK cells. This indicates that the Δg NK cell phenotype could be engineered by knockout of FcRγ. Thus, it would be intriguing to evaluate what role Δg NK cells (or engineered counterparts) play in viremic control of HIV.

Antigen-specific adaptive NK cells mediate recall responses similar to T and B cells and have been described against multiple infectious agents including HIV-1 (128, 140–143). These responses were also activated in humans upon re-exposure to antigens long after their clearance (140). In accordance with the signatures of trained immunity, subsets of antigen-specific memory NK cells have been shown to also undergo epigenetic modifications, resulting in increased chromatin accessibility in regions encoding for genes involved in NK cell activation and function (142). CXCR6 and CD49a are consistently expressed on such antigen-specific memory NK cells and other markers that have been associated with antigen specificity include NKG2D, CD69, CD57, and KLRG1 (128, 140, 141, 143).

NKG2C+ NK cells represent another subset of adaptive NK cells that seem to exert antigen-specific responses. Similar to Δg NK cells, NKG2C+ cells were expanded in people with HCMV infection (144, 145) and influenza vaccination further expands pre-existing NKG2C+ NK cells generated by CMV infection (146). NKG2C recognizes non-classical MHC class I molecules HLA-E. Importantly, NKG2C+ NK cells display antigen-specific responses to HLA-E-binding CMV-derived peptides (147), indicating the potential to engineer NK cell responses by vaccine antigens. Moreover, only 2 HLA-E alleles are mostly represented in humans (148), so HLA-E-mediated NK responses can be triggered regardless of highly polymorphic classical MHC genotypes (149). A recent study demonstrated HLA-E-restricted HIV-1-specific CD8+ T cells responses in PLWH (150), and a CMV-based vector vaccine was able to elicit MHC-E-mediated SIV-specific CD8+ T cell responses associated with protection in a rhesus macaque model (151), further validating that HLA-E-mediated responses are therapeutically inducible. HIV-1 infection also increased the percentage of NKG2C+ NK cells, which is linked to effective viremic control (15). It would be of high interest to determine if HLA-E-dependent HIV-1-specific NK cell responses play a role in HIV-1 control in PLWH. It is important to note that a significant proportion of Δg NK cells also exhibit increased NKG2C expression (136), indicating a potential overlap between these adaptive NK cell subsets. For instance, a considerable percentage of Δg NK cells from PLWH did express NKG2C and their Δg NK cells elicited stronger HIV-1 peptide-specific responses than FcRγ+ NK cells (16).

Because trained immunity is an emerging field of research, limited studies have focused on these responses in HIV-1 ECs or animal models. Nevertheless, considering differential innate immune responses in HIV-1 ECs and the significance of adaptive NK cell responses in PLWH, it would be intriguing to investigate whether trained innate immunity is stronger in individuals with viremic control. Particularly, some aspects of NK cell trained immunity such as antigen-specific adaptive NK cell activities seem to be inducible in a targeted fashion by therapeutic approaches including administration of peptides. Adaptive NK cell functions can also be recapitulated by adoptive transfer of genetically engineered innate cells including CAR NK cells or NK cells with knockout or overexpression of immune genes, so further understanding of NK cell trained immunity could open a new avenue for strategies to elicit immune responses similar to ECs. NK cell trained immunity is also observed in certain animal models, so EC trained immunity elicited by NK cells should be rigorously studied in animal models in order to utilize their advantages such as characterization of innate immunity during well-defined stages of both acute and chronic infection.

## Conclusion

In this review, we highlighted the innate immune responses in ECs characterized in previous studies and reviewed the available animal models to study EC innate immunity. We also discussed the previous and possible future strategies to induce immune responses similar to ECs in other HIV-1 non-controllers by incorporating the novel concept of trained immunity. Because of their association with HLA class I molecules, research on ECs has mainly focused on their CD8+ T cells, frequently neglecting the possibility that innate immunity could also improve their CD8+ T cell functions. While elite control was investigated in humanized mice and NHP models with specific MHC expression profiles, their innate immune responses were understudied. Because ECs represent a limited fraction of PLWH, studies utilizing animal models are essential to meticulously understand their innate immunity. Specifically, humanized mice and SIV-infected NHP models have many strengths including depletion of specific innate immune cells, applying potential immunotherapies with controlled timepoints, and analyzing longitudinal responses including memory responses and trained immunity. These advantages are critical to extensively understand innate immunity in both acute and chronic phases of infection and validate the effectiveness and safety of promising vaccine strategies utilizing trained immunity. Therefore, more extensive research on innate immunity in animal models would be essential to understand the unique innate responses applicable to human ECs that can be translated into novel immunotherapeutics towards other PLWH in a more focused fashion.

## Author Contributions

SS, RR, and SJ conceptualized the manuscript. SS reviewed the literature and wrote the original draft. SJ and RR reviewed and edited the manuscript. All authors contributed to the article and approved the submitted version.

## Funding

This work was supported by the National Institutes of Health (NIH) grants R01 AI116363 (SJ), R01 AI120828 (RR) and R01 AI161010 (RR/SJ) as well as by the NIH-funded BEAT-HIV Martin Delaney Collaboratory to cure HIV-1 infection by Combination Immunotherapy UM1 AI164570.

## Conflict of Interest

The authors declare that the research was conducted in the absence of any commercial or financial relationships that could be construed as a potential conflict of interest.

## Publisher’s Note

All claims expressed in this article are solely those of the authors and do not necessarily represent those of their affiliated organizations, or those of the publisher, the editors and the reviewers. Any product that may be evaluated in this article, or claim that may be made by its manufacturer, is not guaranteed or endorsed by the publisher.

## Abbreviations

ART, anti-retroviral therapy; AIDS, acquired immunodeficiency syndrome; HIV-1, human immunodeficiency virus-1; EC, elite controller; HLA, human leukocyte antigen; SIV, simian Immunodeficiency virus; NHP, non-human primate; MHC, major histocompatibility complex; PLWH, people living with HIV-1; LNTP, long-term non-progressor; VC, viremic controller; IFN, interferon; Th, T helper; NK, natural killer; KIR, killer immunoglobulin-like receptor; ADCC, antibody-dependent cellular cytotoxicity; ILC, innate lymphoid cell; DC, dendritic cell; Cdc, conventional dendritic cell; pDC, plasmacytoid dendritic cell; HAND, HIV-1-associated neurocognitive disorders; MDM, monocyte-derived macrophages; PD-L1, programmed death-ligand 1; GvHD, graft versus host disease; BLT, bone marrow, liver, thymus; HSC, hematopoietic stem cell; AGM, African green monkey; MCM, Mauritan cynomologus macaque ; CAR, chimeric antigen receptor; FcRγ, Fc receptor gamma chain; scFv, single chain portion of the variable domain of antibodies; BCG, Bacillus Calmette-Guérin; △g, Fc receptor gamma chain-deficient; CIML, cytokine-induced memory-like; AML, acute myeloid lymphoma; HCMV, human cytomegalovirus.
